# Evidence for NMR Relaxation Enhancement in a Protic Ionic Liquid by the Movement of Protons Independent of the Translational Diffusion of Cations

**DOI:** 10.1021/acs.jpcb.4c02497

**Published:** 2024-07-05

**Authors:** Magdalena Knapkiewicz, Iga Jankowska, Jolanta Swiergiel, Jadwiga Tritt-Goc

**Affiliations:** Institute of Molecular Physics, Polish Academy of Sciences, M. Smoluchowskiego 17, Poznań 60-179, Poland

## Abstract

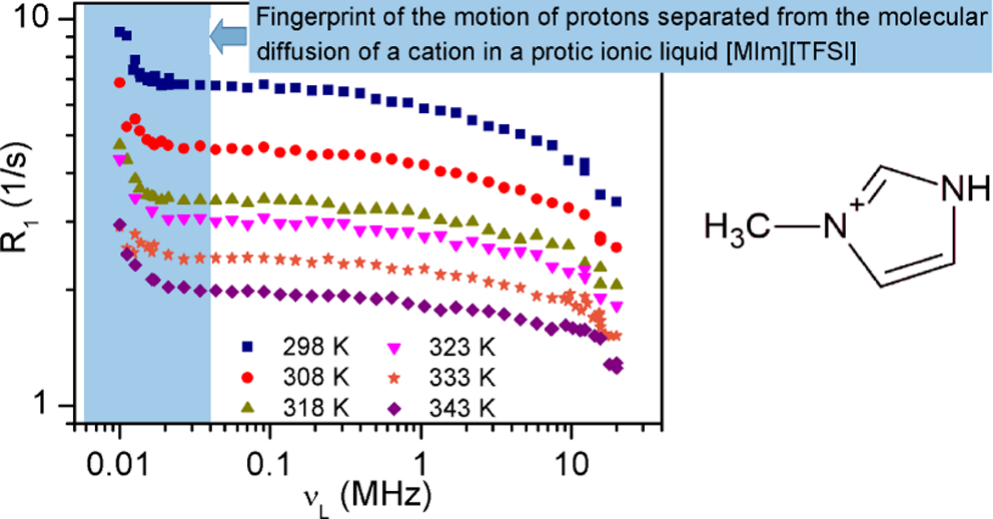

The molecular dynamics, thermal stability, and ionic conductivity were studied in the protic ionic liquid 1-methylimidazolium bis(trifluoromethylsulfonyl)imide ([MIm][TFSI]). The relaxation of the ^1^H spin–lattice of cations in the measured frequency range (10 kHz to 20 MHz) and temperature (298 to 343 K) is sensitive mainly to slow processes occurring in the molecular dynamics of protic ionic liquid and dominated by the contribution of intermolecular translational diffusion. Molecular rotations give only a constant contribution and become significant in the higher frequency range. An interesting feature is the observed enhancement of the ^1^H spin–lattice relaxation below 0.03 MHz attributed to the exchange of protons (order of 10^–5^ s) between imidazolium cations. The measurements of the self-diffusion coefficient of hydrogen atoms of cation from 298 to 343 K additionally confirm the observed phenomenon. The coefficient for exchangeable protons −NH is higher than for the cation. The nuclear magnetic resonance (NMR) experiments provide unambiguous evidence for proton transport decoupled from molecular diffusion of ions and support the conclusion that the charge transport mechanism in the studied PIL includes contributions from both the vehicular and Grotthus mechanisms. The protic ionic liquid is thermally stable to about 573 K as shown by thermogravimetric analysis and its electrical conductivity is 5 × 10^–2^ S/cm at 423 K.

## 1 Introduction

The research conducted in our laboratory is aimed at designing and obtaining new ionically conductive materials for potential applications in ecological energy sources in the intermediate temperature range of 373 to 473 K. We paid great attention to obtaining composites of cellulose and nanocellulose with heterocyclic molecules containing nitrogen. The best electrical conductivity result of about 10^–1^ S/m at a temperature of 413 K under anhydrous conditions, was obtained for nanocrystalline cellulose composite with imidazole.^1^ Unfortunately, the thermal stability of this material is not sufficient for use, e.g., as a membrane in fuel cells. For this reason, we decided to replace imidazole acting as a charge carrier in cellulose composites with another carrier, i.e., a protic ionic liquid (PIL).^2,3^ PILs are a subclass of ionic liquids characterized by low vapor pressure, high thermal stability, and high ionic conductivity, and additionally have an exchangeable proton, usually supported on a cation.^2−4^ The reports of biopolymers with PILs are unique but very promising and were first presented by Danyliv in 2021^5^ and recently by us.^6^ We decided to use protic ionic liquid to overcome the problem of the high viscosity of most “conventional” ionic liquids which makes charge transport usually too slow for electrochemical applications. In PILs, charge transport comes not only from the movement of large ions (translational diffusion of ions) but also from the transport of small and light protons (proton hopping, independent of ionic species’ motions).^7^ In this way, charge transport can be separated from mass transport, unlike in an aprotic ionic liquid, and the conductivity can be increased. We used imidazole-based protic ionic liquids to prepare cellulose/PIL composites because of their higher thermal stability compared to ammonia-based ionic liquids^8^ and immiscibility with water.^9^ The last property is important when we think about the use of ionic liquids in membranes because it prevents the ionic liquid from being washed out of the membrane by water, which is a product of the oxygen reduction reaction at the cathode in the fuel cell, even in the case of an electrolyte that is conductive under anhydrous conditions.

The idea of obtaining a conductive composite with a protic ionic liquid was the inspiration to research the selected ionic liquid 1-methylimidazolium bis(trifluoromethylsulfonyl) imide ([MIm][TFSI]) itself. The cation of this ionic liquid consists of a 5-membrane ring with two nitrogen atoms and three carbon atoms, i.e., a derivative of imidazole, with methyl group substituted on one nitrogen atom and, unlike other members of this type of cations, only one hydrogen on the other nitrogen. Thanks to this active (exchangeable) proton the [MIm][TFSI] is the protic ionic liquid. The exchangeable proton on the cation can form hydrogen bonds to one electronegative atom of the anion (e.g., N, O, or F) or cation. The formation of such a network of hydrogen bonds enables the proton hopping and their transport for a long-range distance unrelated to ion diffusion, i.e., the Grotthuss mechanism.^7^ In general, there are two possible contributions to charge transport in PILs: the movement of ions (anion and cation) and the transportation of small and light protons.^7^ However, whether protons can move faster and independently of cations is a matter of current debate. It is a challenge to provide unambiguous experimental evidence for this process.

The studies of a wide series of 1-alkylimidazolium bis(trifluoromethylsulfonyl)imide ionic liquids with the different alkyl chain lengths (from *n* = 2 to 12) on the imidazolium cation have shown that −NH proton on the cation form stronger H-bonds with the anion for longer alkyl chain and that the ionic conductivity also depends on the alkyl chain length.^10^ The cation-independent transport of protons in PILs has so far been studied using Pulse Field Gradient NMR (PFG NMR) methods,^11^ which allow for the determination of the self-diffusion coefficients of protons of the alkyl chain, imidazolium ring and NH group of PIL.^10,12,13^ The values were very similar for these three types of hydrogen atoms and based on this similarity, a separate movement of the exchangeable protons in the −NH position from the diffusion of the parent cations was excluded in pure PILs. On the other hand, evidence of a proton motion decoupled from molecular diffusion of ions was reported by Martinelli et al.^14^ in the mixture of protic ionic liquid and imidazole but in the low concentration range of imidazole.

Here, we present an investigation of molecular dynamics in 1-methylimidazolium bis(trifluoromethylsulfonyl) imide ([MIm][TFSI]) at the relevant time- and length scales. That was achieved by measuring the diffusion of cations with PFG NMR methods and the proton spin–lattice relaxation time as a function of the magnetic field by the Fast-Field Cycling NMR method (FFC NMR).^11,15,16^ These nuclear magnetic resonance (NMR) methods are the most useful tools in the study of ion dynamics because they are nondestructive, nucleus specific, and allow access to the motions in a broad frequency range through the determination of self-diffusion coefficient, *D*, and spin–lattice relaxation times, *T*_1_. The self-diffusion coefficient provides access to motions in the time scale 1–100 ms and μm length range thus probing the long-range dynamics. The *T*_1_ gives information about molecular motions near the inverse of the resonant frequency. With commercially available spectrometers (e.g., Spin Master, Stelar Mede, Italy) dynamics of liquids can be studied in the broad frequency range between 1 kHz and 40 MHz, referring to the ^1^H resonance frequency but it is possible to go down to 3 Hz through FFC NMR as shown by Fujara et al.^16^ Thus, *T*_1_ relaxation measurement for given temperatures allows probing the short-range dynamics in one single experiment. The *T*_1_ relaxation is measured as a function of the magnetic field but is usually presented as a spin–lattice relaxation rate *R*_1_ (*R*_1_ = 1/*T*_1_) vs Larmor frequency. The plot is named the relaxation dispersion profile or relaxation profile. The obtained relaxation profiles, assuming appropriate theoretical models, will allow determining the correlation times of all types of motions affecting the nuclear relaxation in the PILs. For [MIm][TFSI] we determined parameters characterizing the translational and rotational movements of the cation, but more importantly, we also documented the occurrence of proton motion. The dispersion observed in the relaxation profiles below 0.03 MHz at each temperature studied is due to the slow proton exchange (hopping) between the imidazolium cations. To the best of our knowledge, this is the first evidence of a proton motion decoupled from molecular diffusion of cation in bulk protic ionic liquid.

The article also pays attention to the characterization of the thermal properties and electrical conductivity of 1-methylimidazolium bis(trifluoromethylsulfonyl)imide.

## 2 Materials and Methods

### 2.1 Materials

The studied ionic liquid 1-methylimidazolium bis(trifluoromethylsulfonyl) imide ([MIm][TFSI]) of a purity grade of 98% was purchased from IoLiTec (Ionic Liquids Technologies GmbH). At room temperature, the chemical compound was in the form of a white powder.

### 2.2 Thermogravimetric Analysis (TGA/DTG)

Thermogravimetric analysis and its derivative (TGA/DTG) measurements were investigated using a PerkinElmer TGA8000 apparatus. The measurements were carried out under an N_2_ atmosphere in the temperature range of 303–973 at 10 K/min. The sample of PIL was placed in a platinum pan. Based on the results, were determined: the onset temperature (*T*_onset_) of the decomposition process (5% mass loss) and the maximum decomposition peak (*T*_max_).

### 2.3 Conductivity Measurements

Measurements of the complex dielectric permittivity were performed in the frequency region of 100 Hz–5 MHz by using an HP 4194A impedance/gain analyzer. A homemade measuring capacitor consisted of three plane electrodes (the surface of about 1.2 cm^2^): one central and two grounded on each side, with a distance between them of about 1 mm. The shape of the capacitor electrodes is rectangular and they are made with a gold-plated copper. The probing electric field intensity, *E*, was equal to about 1 V/mm. The measurements were performed in the temperature range from 298 to 423 K. At first, the samples were cooled down from 343 to 295 K and next the measurements were performed for increasing temperature. The temperature of the measuring cell was controlled with a “Scientific Instruments” device, model 9700, within ±2 × 10^–3^ °C.

### 2.4 NMR Experiments

Proton NMR spin–lattice relaxation measurements were performed on a SpinMaster NMR relaxometer from Stelar Company (Mede, Italy) with a *B*_0_ magnetic field of 0.5 T. The system enables measurements of relaxation profiles of samples from 10 kHz to the maximum operating magnetic field of 20 MHz (^1^H Larmor frequency). Below 10 MHz, the prepolarization sequence with the polarizing magnetic field corresponding to 20 MHz was applied for a time of 5*T*_1_. Above 10 MHz, the nonpolarized sequence was used. For each resonance frequency, 16 magnetization values versus time in a logarithmic time scale have been recorded. The switching time of the magnet was set to 3 ms. More details on the FFC NMR technique can be found elsewhere.^11,15,16^ The measured NMR signal comes only from the [MIm]^+^ cation because the anion of the tested liquid does not contain hydrogen atoms and turned out to be single-exponential for all measured temperatures. Thus, the single *T*_1_ relaxation times were calculated with an error of about 5%. ^1^H-T_1_ relaxation times of [MIm][TFSI] were studied in the temperature range from 298 up to 343 K (*T*_m_ = 328 K). The temperature of the samples was controlled to an accuracy better than 0.5 K. The samples without drying were used for NMR measurements. The powder was transferred directly from the manufacturer’s vial to the NMR tube and sealed immediately. We assume that the water content, if any, was very low because the sample’s high-resolution ^1^H NMR spectra (Figure 7) did not show any detectable peaks other than the main peaks assigned to the PIL.

The additional *T*_1_ measurements at a proton resonance frequency of 500 MHz were performed with a Bruker Avance III HD spectrometer for all studied temperatures. This spectrometer was also used to carry on the pulse-field gradient-type diffusion experiments. The self-diffusion coefficient for the cation of [MIm][TFSI] was obtained with a stimulated echo pulse sequence and calculated by fitting the Stejskal-Tanner equation^17^ to the signal’s intensity attenuation data according to
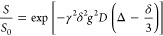
1where *S* and *S*_0_ are echo signal intensities, with and without magnetic field gradient pulse applied, respectively, γ is the gyromagnetic ratio, δ is the gradient pulse duration, and *D* is the self-diffusion coefficient. The measurements were carried out as a function of the magnetic field gradient strength, *g*, which changed in 32 steps, and the parameters δ and Δ were equal to 1 and 20 ms, respectively, and remained unchanged during the measurements. The measurements were performed for the same temperatures as the *T*_1_ measurements.

## 3 Theory of Spin-Lattice Relaxation

Oscillating electromagnetic fields near nuclear spins are crucial for NMR transitions between spin energy levels. They arise from interactions between spins and the random motions of molecules, which causes the interactions to fluctuate in time. From the theory of nuclear magnetic relaxation in ionic liquids, the most important interactions responsible for the relaxation processes are direct dipole–dipole interactions between nearby nuclear spins^11,18−21^ although Coulombic interactions, along with van der Waals, hydrogen bonds, and π–π interactions dominate between IL ions.

According to spin relaxation theory, relaxation rates are linear combinations of spectral density functions which are defined as the Fourier transform of the corresponding correlation functions that characterize the dynamic processes leading to stochastic fluctuations of dipole–dipole magnetic interactions and thus causing relaxation processes.^11,18−21^ The mathematical form of the correlation function and therefore of the spectral density depends on the mechanism of the motion. Therefore, relaxation dispersion profiles, i.e., the relaxation rate of the spin–lattice as a function of the resonance frequency, are a direct fingerprint of this mechanism.

The relaxation dispersion in bulk ionic liquids can be fully described by models taking rotational and translational motions into account. One advantage of NMR relaxometry data analysis is its possibility to separate these contributions, and the total relaxation rate can be written as a sum of these processes
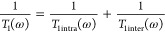
2where ω_0_ = 2π*ν*_0_ = γ*B*_0_ denotes the proton Larmor frequency in the magnetic field *B*_0_ and γ is the gyromagnetic factor. The *T*_1intra_ expresses the intramolecular magnetic dipole interactions among protons within the same molecules (ions) modulated by local molecular reorientations. The contribution of *T*_1inter_ relaxation comes from intermolecular magnetic dipolar proton interactions (cation–cation and/or cation–anion), which fluctuate in time as a result of the relative translational self-diffusion of molecules.

The relaxation rate of the homonuclear spin–lattice for intramolecular dipolar interactions, 1/*T*_1intra_, is given by the Bloembergen, Purcell, and Pound equation,^18^ which takes the following form, assuming the Lorentz form of the spectral density
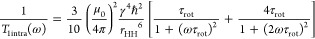
3where *τ*_rot_ denotes a single rotational correlation time characterizing the fluctuation of the dipole–dipole interactions, μ_0_ is the magnetic susceptibility of vacuum, ℏ = *h*/2π where *h* is the Planck constant, and *r*_HH_ is the intramolecular distance. The parameters before the bracket define the dipole–dipole interaction constant, marked with the symbol *D*_intra_. The homonuclear intermolecular contribution to the spin–lattice relaxation, 1/*T*_1inter_, is given by the following equation^18^
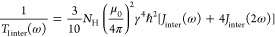
4where *J*_int*er*_(ω) is the translational spectral density function,  where *n*_H_ is the number of nuclei per unit volume, *N*_A_ is the Avogadro number, ρ is the density of the ionic liquid, and *M* is the molecular mass. The most commonly used model to describe the effect of translational diffusion on the spin–lattice relaxation time is the force-free hard-sphere model^20,21^ with *J*_int*er*_(ω) given as
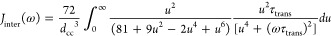
5where *u* is an integration variable, *d*_cc_ is the distance of the closest approach of interacting species, and  is the translational correlation time. The parameter *D*_12_ is a relative translational diffusion coefficient expressed as the sum of the self-diffusion coefficient of the interacting ions *D*_12_ = *D*_1_ + *D*_2_. In the present case, identical ions (cations) are interacting and thus the final relation is *D*_12_ = 2*D*. Equation 4, after inserting eq 5, can be rewritten as
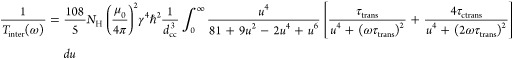
6In the protic ionic liquid, [MIm][TFSI], only the MIm^+^ cation contains ^1^H nuclei, and only TFSI^–^ anion contains ^19^F nuclei. Therefore, we can independently obtain information about the dynamics of ions. However, only the cation dynamics are the subject of our research. The investigation on imidazolium-based PILs with anion containing ^19^F showed that in the case of proton spin–lattice relaxation, the heteronuclear translational contribution of fluorine anions can be neglected^22−24^ and the ^1^H relaxation dispersion can be described taking into account only the intra- and intermolecular interactions of protons.

The contribution, 1/*T*_1ex_, to the total relaxation rate due to proton exchange^16,25^ gives a Lorentz-shaped term
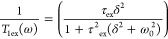
7where the τ_ex_ is the proton exchange time and δ^2^ is the mean quadratic interaction strength between the ^1^H and interacting spins.

The total proton spin–lattice relaxation rate 1/*T*_1total_, of studied PIL consists of a relaxation rate, of 1/*T*_1intra_, due to the intramolecular reorientations of cations, 1/*T*_1inter_ due to the translational diffusion of cations, and 1/*T*_1ex_ due to the proton exchange
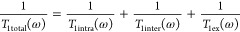
8After inserting the appropriate relaxation contributions given by eqs 3, 6, and 7 into eq 8, we obtain the final form of the equation that was used to analyze the experimental relaxation dispersion profiles.

## 4 Results and Discussion

### 4.1 Thermal Properties

**Figure 1:**
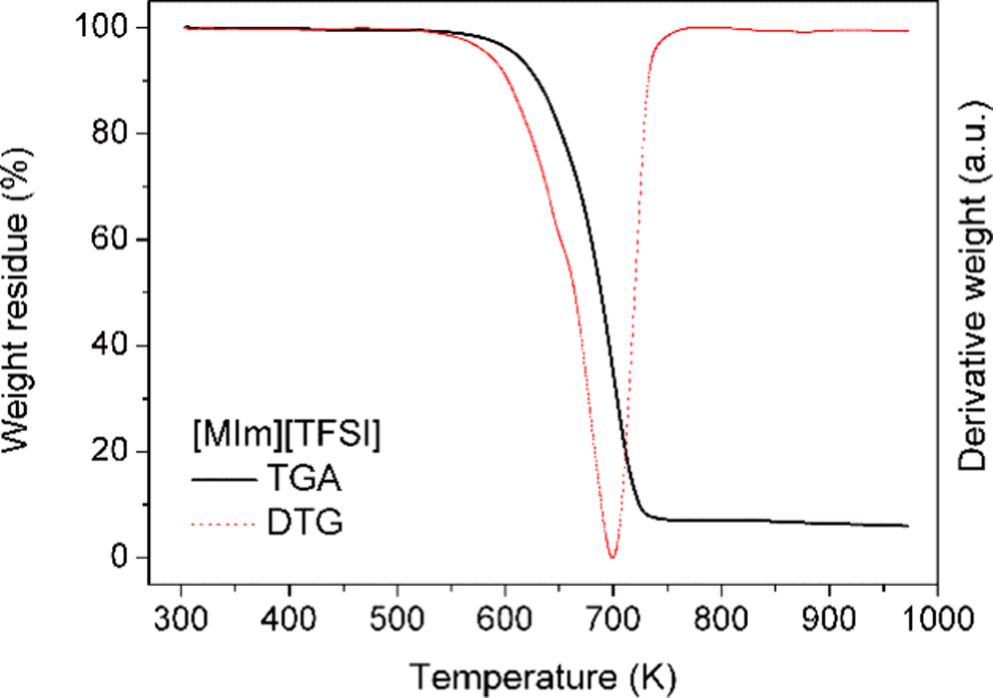
TGA thermogram with the corresponding derivative DTG for pure [MIm][TFSI] measured at a 10 K/min heating rate.

The thermal stability of the [MIm][TFSI] was investigated by thermogravimetric analysis, which provides information on the mass loss of materials with increasing temperature. Figure 1 depicts the TGA thermogram and its corresponding DTG curve for [MIm][TFSI]. PIL is thermally stable to about 573 K. The process of decomposition of [MIm][TFSI] displays a mass loss step above 573 K, with a maximum decomposition temperature of 698 K. The onset decomposition temperature is 583 K. The temperature maximum for [MIm][TFSI] is similar to other alkyl-imidazolium salts.^26^

### 4.2 Ionic Conductivity

**Figure 2:**
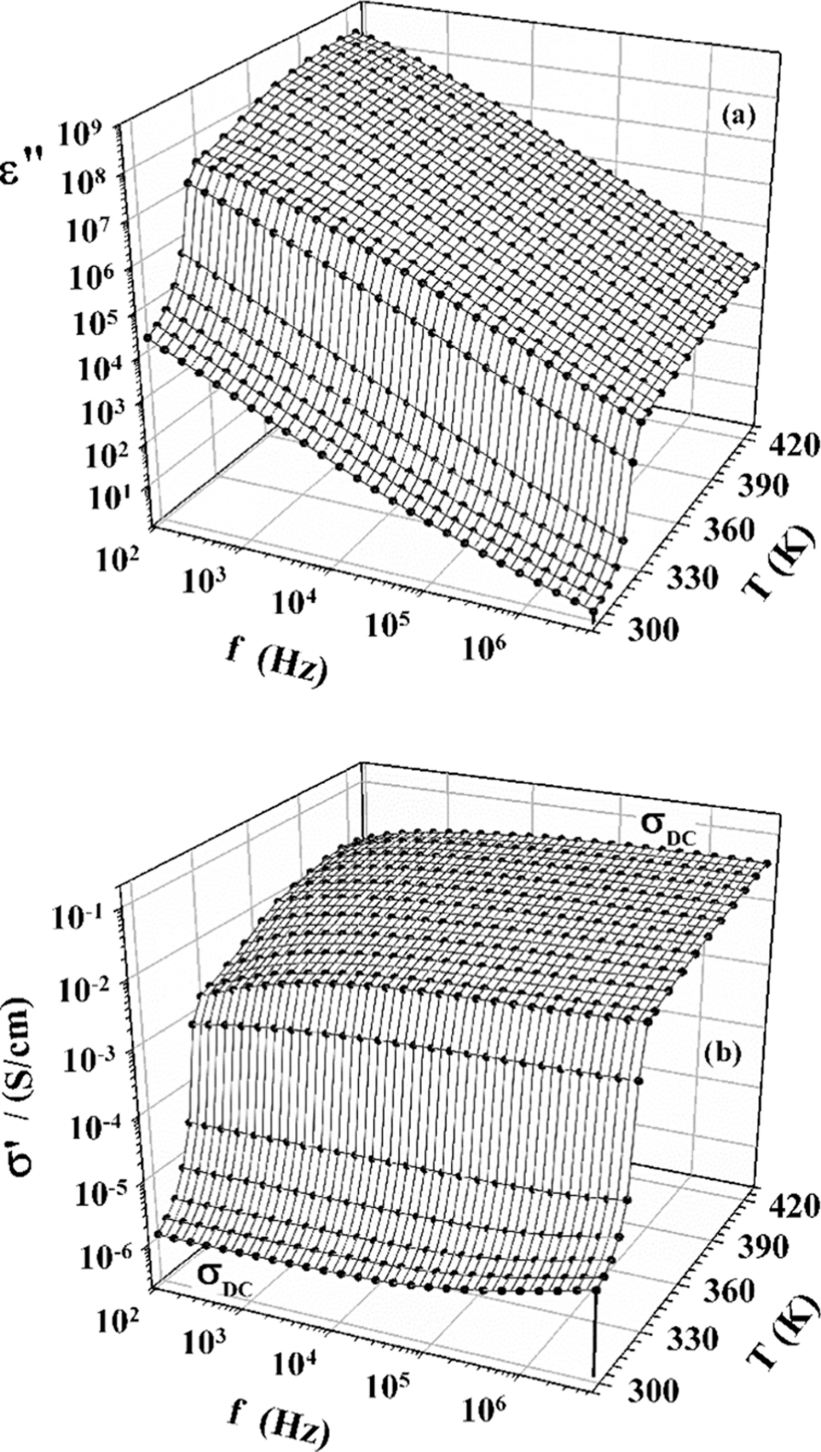
Imaginary part of the dielectric spectra [MIm][TFSI] as a function of frequency (*f*) for different temperatures (a) and the corresponding real part of conductivity spectra (b).

Figure 2a presents the imaginary part of the dielectric spectra, ε″, of [MIm][TFSI] recorded in the frequency range from 100 Hz to 5 MHz, at different temperatures, and in solid and liquid phases. In the temperature range studied, the PIL exhibits the solid-to-liquid phase transition. The melting process in the PIL manifests itself as a significant change in the character of the permittivity temperature dependence.

The imaginary part of the dielectric spectrum, for low enough frequencies in comparison to those where the maximum of the dielectric loss of the studied liquid occurs, can be expressed by reduced Debye equation: ε″ = σ_DC_/ωε_0_ where σ_DC_ is called a direct current ionic conductivity. According to this relation, ε″(see Figure 2a) should present straight lines of slope −1 (on a log–log scale). The data ε″ presented in Figure 2a can be transformed into the real part of conductivity spectra, σ′, according to the relation where ε_0_ = 8.85 pF/m is the permittivity of free space, see Figure 2b. The value of σ_DC_ was calculated from the linear dependence of log ε″ vs log *f* which occurs in the high-frequency range (from about 25 kHz to 5 MHz) above 323 K and in the low-frequency range (from about 100 Hz to 15 kHz) in the temperature range of 298–318 K. For real samples, some deviation from Ohm law is observed as seen in Figure 2 at low frequencies for high temperatures and at high frequencies for low temperatures. The decrease in the slope of the logε″ vs. logf dependence observed in the low frequencies (for high temperatures) manifests itself as a decrease in the electric conductivity of [MIm][TFSI]. The effect is a consequence of the double-layer formation near the blocking electrodes of the measuring capacitor.^27^ The increase of σ′(f) observed at higher frequencies (for low temperatures) is usually associated with relaxation processes. In the case of ionic liquids, these may be reorientation movements of ions.^28^

**Figure 3:**
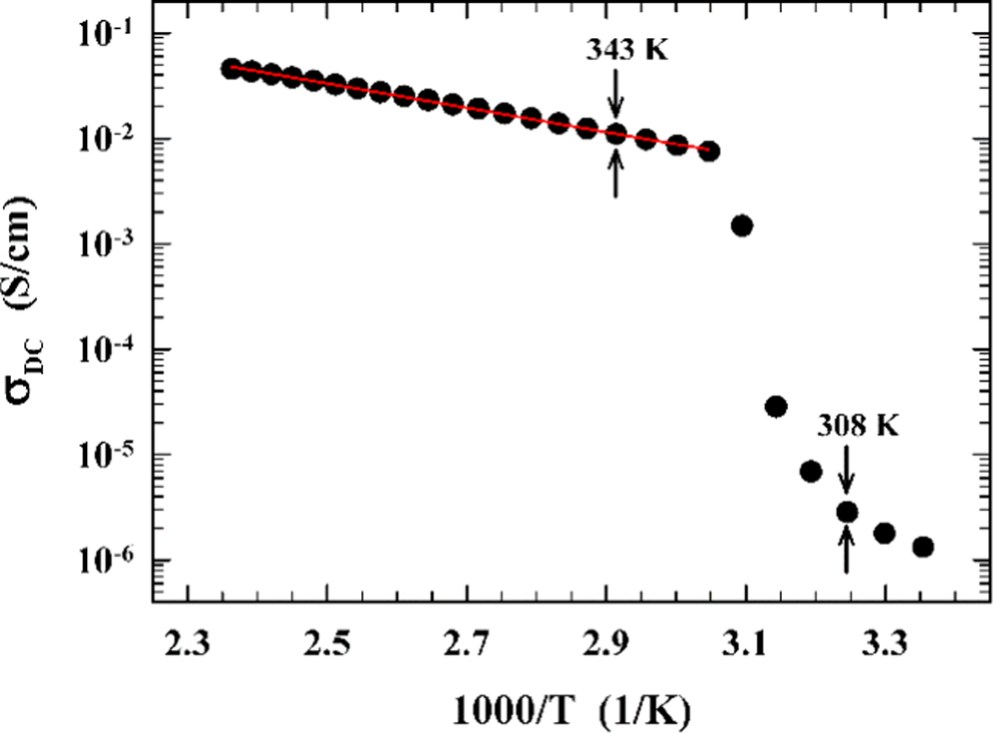
Temperature dependence of the dc-conductivity for the protic ionic liquid [MIm][TFSI]. The figure also shows the temperatures for which a full analysis of the dispersion profiles of NMR relaxation times was performed.

The physical quantity σ_DC_ obtained in our experiment for solid and liquid phases in [MIm][TFSI] is presented in Figure 3 as a function of temperature. A clear solid–liquid transition is visible at 328 K. This result is in line with previously reported by Abdurrokhman^10^ that only the PILs with very short or very long alkyl chains have clear solid–liquid transitions. The studied PIL shows the highest conductivity among this type of PILs and confirms the previously demonstrated relationship that the conductivity decreases with the increase of the alkyl chain.^10^

### 4.3 Cation Dynamics

A series of ^1^H NMR spin–lattice relaxation times were measured for the cation of the protic ionic liquid [MIm][TFSI] in the temperature range from 298 to 343 K.

**Figure 4:**
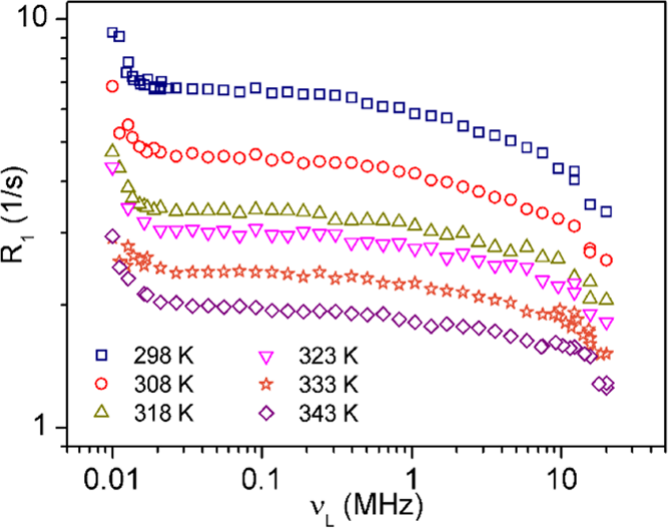
^1^H relaxation dispersion profiles for [MIm]^+^ cation in the temperature range from 298 to 343 K.

The temperature range was selected to cover the solid and liquid phases of the PIL and the transition region. The relaxation dispersion profiles are shown in Figure 4 as a function of the external magnetic field expressed in the Larmor frequency units.

**Figure 5:**
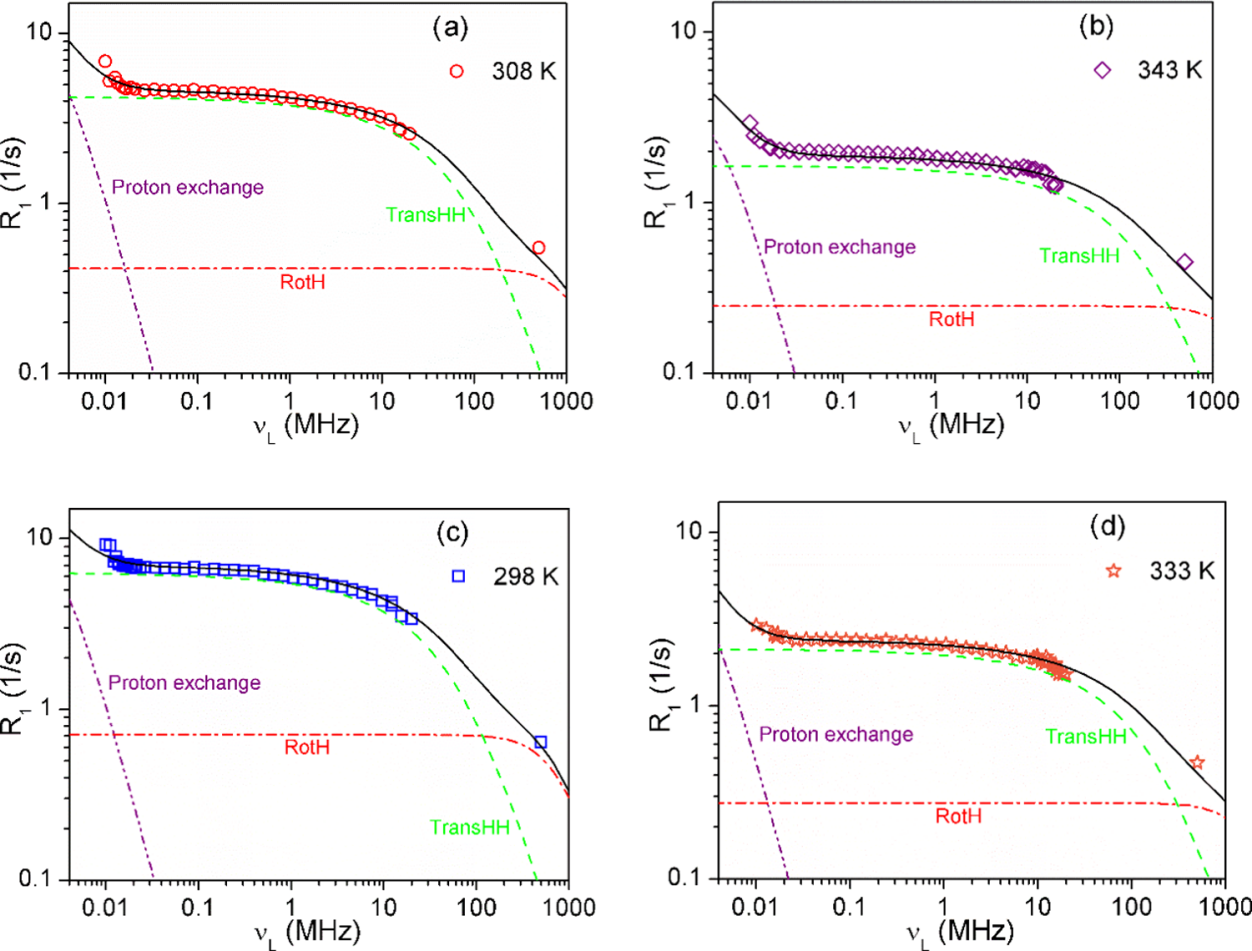
^1^H relaxation dispersion profiles for [MIm]^+^ cation in a solid (a, c) and liquid phase (b, d).

The relaxation rate, *R*_1_, strongly depends on the temperature and becomes shorter when the temperature increases. A significant increase in *R*_1_ is observed in all dispersion profiles below 0.03 MHz. Above this value, *R*_1_ gradually decreases as the Larmor frequency increases. No indication of sudden changes in the relaxation value was observed, which would be a signature of the phase transition process from the solid to the liquid state (328 K). The same applies to the shape of the relaxation dispersion profile, which remains similar in both phases. Figure 5a,b presents a sample fitting to the experimental data collected at selected temperatures, showing the separate contributions to the ^1^H spin–lattice relaxation. The experimental data also contained the R_1_ values measured at 500 MHz. These data were of great importance to the analysis of the results because they allowed for a more faithful reproduction of the shape of the relaxation profile. The spin–lattice relaxation in the entire range of studied frequencies was analyzed based on the model given by eq 8. The solid lines in Figure 5 present the best fit of the model to the experimental data, while the dashed lines reflect the contribution of individual relaxation mechanisms. We used a web-based solution for professional model fitting^29^ created and described by Sebastiao^30^ to analyze the data.

The solid lines represent the best fit of eq 8 to the experimental points with the fitting parameters given in Table 1. The dashed lines represent contributions due to translational diffusion, rotational dynamics, and proton exchange.

**Table 1:** Parameters characterizing, the rotational, τ_rot_, and translational dynamics, τ_trans_, and proton exchange, τ_ex_, between cations [MIm]^+^ along with the distance of the closest cation approach, *d*_cc_.^a^

*T* (K)	τ_rot_ (s)	*d*_cc_ (m)	*τ*_trans_ (s)	*τ*_ex_ (s)
298	1.06 × 10^–10^ (±0.13 × 10^–11^)	4.95 × 10^–10^ (±0.10 × 10^–10^)	5.94 × 10^–9^ (±0.01 × 10^–10^)	5.18 × 10^–5^ (±0.37 × 10^–5^)
308	6.21 × 10^–11^ (±0.47 × 10^–11^)	4.95 × 10^–10^ (fixed)	4.01 × 10^–9^ (±0.02 × 10^–10^)	5.11 × 10^–5^ (±0.14 × 10^–5^)
333	4.10 × 10^–11^ (±0.13 × 10^–11^)	0.95 × 10^–10^ (fixed)	2.01 × 10^–9^ (±0.01 × 10^–10^)	6.41 × 10^–5^ (±0.15 × 10^–5^)
343	3.69 × 10^–11^ (±0.04 × 10^–11^)	4.95 × 10^–10^ (fixed)	1.56 × 10^–9^ (±0.55 × 10^–10^)	3.32 × 10^–5^ (±0.09 × 10^–5^)

a The 298–308 K temperature range covers the PIL solid phase, while the 333–343 K range covers the liquid phase.

In the studied frequency range, the relaxation is dominated by homonuclear intermolecular relaxation due to translational diffusion (H–H). In agreement with recent investigations on imidazolium-based ILs with anions containing ^19^F, the contribution due to heteronuclear relaxation (H–F) for ^1^H can be ignored.^22−24^ Generally, ^1^H relaxation rates also include contributions due to ^1^H–^19^F dipole–dipole interactions modulated in time by the relative translational diffusion of ions. We are aware that not considering this contribution in the analysis of dispersion curves is a simplification, but taking them into account means the need to determine three additional parameters in the fitting procedure. The experimental data were very well reproduced without the contribution of ^1^H–^19^F relaxation, as seen in Figure 5, which justifies our simplification. The contribution of molecular rotations which modulate the intramolecular interactions gives only a constant contribution and becomes significant in the higher frequency range. At the lowest tested frequencies, below 0.03 MHz, the influence of an additional relaxation mechanism becomes visible. Unfortunately, we could only study this process in the narrow frequency range due to hardware limitations. We postulate that the origin of the relaxation enhancement observed at lower frequencies is due to the magnetic interaction of ^1^H–^14^N modulated by the slow process of proton exchange (proton transfer) between imidazolium cations: a proton from one cation (−NH proton) “visits” ^14^N on the side of another cation and leads to the local fluctuation of the field, which gives a contribution to the spectral density at low frequencies. The concept of pairing ions with opposite charges is well-accepted in ionic liquids. The association of like-charged ions seems unlikely. However, there is already experimental evidence supporting such possibilities. Direct spectroscopic evidence for H-bonded cation–cation clusters similar to those known for water and alcohols was reported for the ionic liquid, 1-(2-hydroxyethyl)-3-methylimidazolium tetrafluoroborate.^31^ Mele et al. reported NOE contacts between protons of imidazolium cations.^32^ A gradual self-association of the cations was also observed while studying the structure and properties of 4-oxopiperidinium salts [OC5 H8 NH2]X for a series of anions X(−) of decreasing basicity.^33^

To eliminate the number of fitting parameters in eq 8, we calculated the value of the *N*_H_ parameter, which is 8.07 × 10^28^ m^–3^. Based on the equilibrium geometry of a single imidazolium cation and using the relation  were *r*_IS_ the distance between all protons within the cation and *N*(*N* – 1)/2 is the number of IS pairs within the cation, the intramolecular distance *r*_HH_ of 2.14 × 10^–10^m was calculated, which gives the *D*_intra_ constant in eq 3 of 1.34 × 10^9^ Hz^2^. The best-fitting parameters of eq 8 to the experimental data from Figure 5 are collected in Table 1.

The proposed NMR relaxation model fits well with the experimental relaxation profiles and the values of parameters *τ*_rot,_*τ*_trans_, and *d*_cc_ are consistent with those obtained for similar cations.^10,12,23,24^ The parameter *τ*_ex_, can be compared with previously published data for proton exchange in different materials. To our knowledge, proton exchange by the FFC NMR method has only been evidenced in water as dispersion of the proton spin–lattice relaxation time observed below 10^4^ Hz.^16,25^ It was assumed that the origin of this dispersion is due to the slow exchange of protons between different oxygen environments, which modulates the magnetic ^1^H–^17^O interaction. The fitted parameter *τ*_ex_ was equal to 3.4 × 10^–4^ s at 296 K and 1.4 × 10^–4^ s at 353 K.^16,25^ In our case *τ*_ex_ is the order of 10^–5^ s. This means that the proton exchange process in the tested PIL is faster than in water and thus contributes to relaxation at higher frequencies than in water. The proton exchange time in studied PIL correlates well with the value estimated for proton exchange between imidazole in imidazole-doped cellulose, obtained from the analysis of the line shape of solid-state NMR spectra.^34^

So far, experimental evidence for cation-independent proton transport in PILs comes mainly from measurements of the self-diffusion coefficient of cation hydrogen atoms by PFG NMR.^12,13^ Such evidence is considered to be a higher *D*_NH_ value measured for the exchangeable proton than for other protons or the cation as a whole.

**Figure 6:**
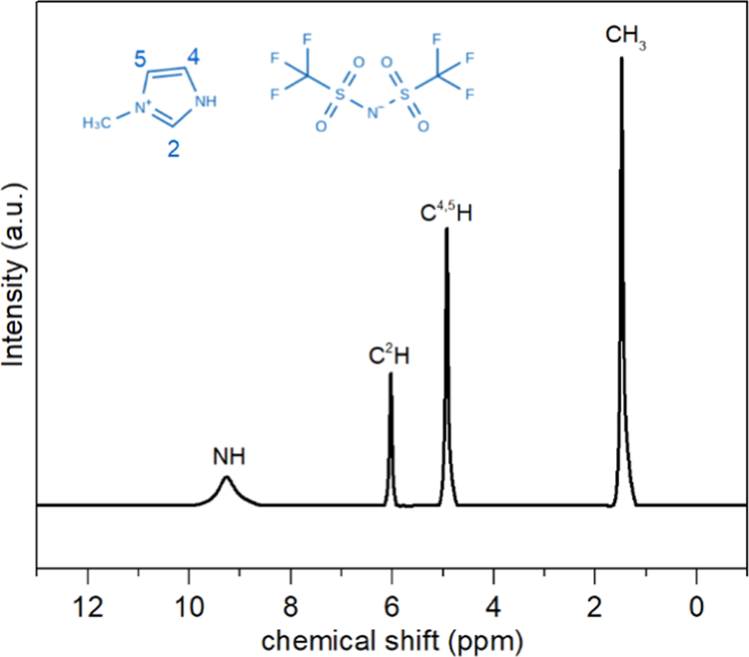
Liquid state ^1^H NMR spectrum of [MIm]^+^ cation in the [MIm][TFSI] protic ionic liquid. The NMR peak assignment is consistent with the labeling in the PIL molecular structure in the figure.

The 1D proton NMR spectrum of [MIm][TFSI] at 343 K (liquid phase) along with the chemical structure and peak assignment is shown in Figure 6.

**Figure 7:**
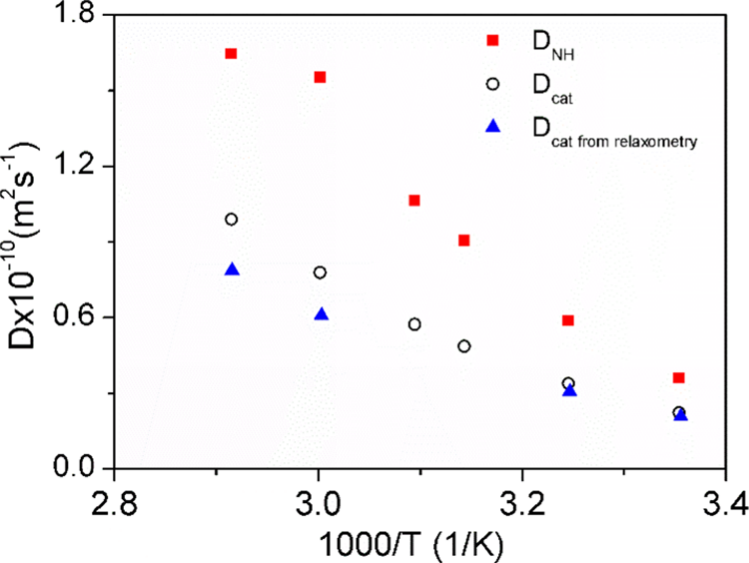
Self-diffusion coefficients for [MIm]^+^ cation (*D*_cat_) and the exchangeable NH proton (*D*_NH_) were measured as a function of temperature by PFG NMR. For comparison, the *D*_cat_ values calculated from NMR relaxometry were added at temperatures of 298, 308, 333, and 343 K.

It is easy to see that the signal of the exchangeable proton −NH is broader than the signal of the nonexchangeable protons from C_2_H, C_4_H, and C_5_H. Separate ^1^H NMR resonances from Figure 6 were used to calculate the self-diffusion coefficient of the cation as whole molecules, *D*_cat_, which is the average of the values obtained from the ring protons. The *D*_NH_ is calculated from the resonance assigned to the NH groups. The values of *D*_cat_ and *D*_NH_ show a similar temperature dependence, with *D*_NH_ values becoming increasingly higher than *D*_cat_ with increasing temperature (see Figure 7). The larger *D*_NH_ than *D*_cat_ can be explained by assuming that in [MIm][TFSI] protons diffuse faster than the [MIm] cation. This means that *D*_NH_ contains a contribution from *D*_cat_ and *D*_H_, where *D*_H_ is the proton self-diffusion coefficient. Therefore, the charge transport mechanism in the studied PIL includes contributions from both the vehicular and Grotthus mechanisms. Our results are contrary to those obtained for other protic ionic liquids based on imidazolium and triazolium^13^ where the reported values of the self-diffusion coefficients were very similar for all hydrogen atoms, which indicated the diffusion of an exchangeable proton together with the cation.

It is worth comparing the translational self-diffusion coefficient of the cation obtained directly from PFG NMR with those obtained indirectly from the relaxation data analysis. We calculated the relative diffusion coefficient of [MIm] cation in ([MIm][TFSI] taking into account parameters characterizing the translational dynamics of the cation (*τ*_trans_ and *d*_cc_ from Table 1) and the relationship . In the case of uncorrelated motion, the relative diffusion coefficient is given as a sum of the self-diffusion coefficient (measured by NMR gradient methods) of interacting ions *D*_12_ = *D*_1_ + *D*_2_) in our case cations of studied PIL. Thus, for identical cations [MIm] the relative diffusion coefficient is twice as large as the self-diffusion coefficient (*D*_12_ = 2*D*_cat_). The obtained values are denoted as *D*_cat from relaxometry_ (being, in fact, equal to half of the relative translational diffusion coefficient) and plotted in Figure 7.

For a given temperature, the *D*_cat_ values from relaxometry should be equal to the *D*_*c*at_ values from PFG NMR, assuming no correlation in cation dynamics. In Figure 7, the values agree well at lower temperatures (298 and 333 K) but differ at high temperatures (333 and 343 K)—the values from the diffusometry measurements are larger. This discrepancy may suggest that the relative cation–cation translational motion becomes more correlated with increasing temperature.^35^

## 5 Conclusions

The studied PIL is thermally stable up to approximately 573 K and its electrical conductivity is of the order of 5 × 10^–2^ S/cm at 423 K. Such parameters are satisfactory if we take into account the possibility of using this ionic liquid to obtain membranes, among others, for fuel cells operating in the intermediate temperature range of 373 to 473 K. For this temperature range, there are still no efficient membrane materials and in this respect, PIL has the potential to fill this gap.

We believe that the most interesting result of the presented research concerns the dynamics of the ionic liquid. In the range of magnetic field strengths, where the presented measurements were made using the FFC NMR method, it was shown that NMR relaxation is sensitive mainly to slow processes occurring in the molecular dynamics of protic ionic liquid [MIm][TFSI]. The relaxation is dominated by the contribution of intermolecular translational diffusion, enhanced at the lowest magnetic field by an additional mechanism, i.e., proton exchange between slowly diffusing cations. To date, most of the reported experimental evidence for cation-independent proton transport comes from PFG NMR measurements of the self-diffusion coefficient of hydrogen atoms. Based on this method, no separate movement of exchangeable protons in the −NH position from the diffusion of parent cations was detected for any of the protic ionic liquids studied so far.^10,12,13^ Therefore, we believe that the observed low-frequency dispersion of the spin–lattice relaxation times of cationic protons is the first experimental evidence of this phenomenon in PILs. The observation of independent proton transport in PIL was possible due to the sensitivity of the FFC NMR method. The observed phenomenon was additionally supported by measurements of the self-diffusion coefficient using the PFG NMR method. The measured *D*_NH_ for the exchangeable protons was higher than for the cation as a whole. Finally, we can conclude that NH protons can diffuse via the Grotthuss mechanism and therefore faster than the cationic molecule. Thus, the charge transport mechanism in the studied PIL includes contributions from both the vehicular and Grotthus mechanisms.
